# Deep Learning Methods Capture Non-Linear Brain Aging Patterns Underlying Alzheimer’s Disease and Resilience

**DOI:** 10.1093/geroni/igab046.1436

**Published:** 2021-12-17

**Authors:** Kyra Thrush, Albert Higgins-Chen, Yaroslav Markov, Raghav Sehgal, Morgan Levine

**Affiliations:** Yale University, New Haven, Connecticut, United States

## Abstract

The current era of multi-omics data collection has enabled researchers to obtain exceptionally comprehensive profiling of disease subjects. However, exceptionally high dimensionality can ultimately be an obstacle to biological insight. Previously, we presented a method in which penalized regression of methylation principal components reduces noise and improves prediction of age, disease, and Alzheimer’s Disease (AD) pathophysiology. However, strictly linear methods may overly simplify the complex epigenetic aging landscape. We hypothesized that non-linear deep learning methods could identify molecular signatures that better reflect individual resilience to AD. Through the use of an autoencoder to represent high dimensional methylation array data, and supplemental machine learning methods, we connect latent nonlinear representations of the brain to aging, resilience, and indications of AD. In particular, resultant age-predicting representations of methylation were correlated with enrichment of methylation regions and biological pathways. Contextualized within AD pathology, this work provides valuable, ongoing insight into resilience in AD.

